# Quality of life, social support, and adherence in female patients with thyroid disorders

**DOI:** 10.1186/s12905-023-02718-0

**Published:** 2023-11-02

**Authors:** Eliza Kollerits, Ágnes Zsila, Balázs Matuszka

**Affiliations:** 1 ELTE Doctoral School of Psychology, Kazinczy u. 23–27, Budapest, 1075 Hungary; 2 ELTE Institute of Psychology, Kazinczy u. 23–27, Budapest, 1075 Hungary; 3 Institute of Psychology, Pázmány Péter Catholic University, Mikszáth Kálmán tér 1., Budapest, 1088 Hungary

**Keywords:** Adherence, Quality of life, Social support, Thyroid disorders, Women’s health

## Abstract

**Background:**

According to the 2010 European Health Interview Survey, 51% of women in Hungary have a chronic disease, and is among the poorest quartile in the EU countries. Thyroid diseases affected more than 650,000 women in 2021 based on a recent report by the Hungarian Central Statistical Office. Despite the high prevalence rates, quality of life in these patients is scarcely researched in Hungary. To fill this gap, this study aims to explore the associations of the quality of life of thyroid patients in Hungary with social support and adherence.

**Methods:**

A cross-sectional study was conducted via an online questionnaire. Data from 885 female Hungarian thyroid patients with pharmacological treatment (M = 35.6 years, SD = 10.7, age range: 18–73 years) were analyzed. Participants were divided into two patient groups based on the type of thyroid disorder: hypothyroidism (*n* = 824; 93.1%) and hyperthyroidism (*n* = 61; 6.9%). Group comparisons, correlations, and a mediation model were performed to explore differences between thyroid patients.

**Results:**

No differences were found between patients with different types of thyroid disorders in quality of life, adherence, and social support. Consistent, weak associations were found between quality of life and social support in both patient groups. Higher perceived social support partially explained the relationship between adherence and life quality in thyroid patients.

**Conclusions:**

No substantial differences were found between patients with different types of thyroid disease in mental well-being indicators. These patients are psychologically more vulnerable and need a socially supportive environment to recover, because higher adherence is associated with a better quality of life, and social support can facilitate this process.

## Introduction

According to the report by the 2010 EuroVaQ, the overall health status of the Hungarian population is poorer than the other European countries which were included [1] and is among the poorest quartile in the EU countries [2]. In 2019, 48% of the Hungarian population had a chronic disease, affecting nearly 5,000,000 people [3]. Moreover, 51% of women had a chronic disease in Hungary [3]. Some chronic diseases are surveyed in Hungary (e.g. psoriasis, rheumatoid arthritis, multiple sclerosis, epilepsy, overactive bladder, asthma, COPD, Parkinson's disease, dementia, diabetes, PCOS, endometriosis) [4–15]. However, the population of people living with thyroid disorders is scarcely investigated (e.g., [6, 16–18]). The prevalence and incidence rates of disorders based on abnormal thyroid functioning have increased between 1999 and 2021 in Hungary [19]. Even though thyroid disorders can be considered as a public health problem, relatively little attention has been paid to the psychological well-being of these patients in Hungarian research practice. However, the endocrine system, including the thyroid gland, influences a wide range of psychological and somatic mechanisms (e.g., stress level, attention) [20–25].

Psychological mechanisms were investigated predominantly in psychiatric disorders. Previous studies have found evidence for the association between thyroid disorders and mental health [23–31]. Hypothyroidism has been associated with depression [23, 24, 26–28], anxiety [28], bipolar and borderline personality disorder [25, 32]. Similarly, patients diagnosed with Hashimoto's thyroiditis are more likely to develop depression, anxiety, or borderline personality disorder [29, 30, 33]. Hormone markers also predicted the development of unipolar and bipolar depression [34, 35]. Hyperthyroidism was also associated with anxiety [31], bipolar disorder [36]. Additionally, subclinical hyperthyroidism was associated with depressive symptoms [37, 38]. People with hyperthyroidism have increased rates of hospitalization for psychiatric diagnoses both before and after being diagnosed with hyperthyroidism [39]. For instance, women with Graves' disease are at a higher risk for mood disorders and anxiety [36, 40], and depression [41]. Mental health claims as an important role in the course of thyroid disorders.

### Quality of life in thyroid patients

The quality of life can be affected by the symptoms experienced by patients diagnosed with Hashimoto's thyroiditis [42], most commonly by mood disturbances and fatigue [43, 44]. There is evidence for the deterioration in life quality, which may not even correlate with hormone levels [43–45]. Regulating hormone levels with medication may not be sufficient to manage all the symptoms [46]. Most studies on life quality investigated the effectiveness and impact of hormone replacement, other medication, and surgical removal of the thyroid gland. The effectiveness of hormone replacement has been demonstrated; however, this may also involve many indirect psychological effects. For instance, the normalization of metabolic rate, because of the hormone replacement, can lead to weight loss in hypothyroidism, which improves the quality of life of patients [44, 47–49].

Hyperthyroidism was associated with mood disorders [36] sexual disfunctions [32] and reduced quality of life in adults [39, 50]. Some specific symptoms (e.g., Graves-Orbitopathy) can have a severe negative impact on life quality as these symptoms may alter physical appearance (e.g., look and face) [51]. For this purpose, specific health-related quality of life measures are commonly used with thyroid patients [17, 51, 52]. Overall, several studies have demonstrated that all kind of thyroid patients are struggling with psychological challenges, which affects their quality of life and their approach to their condition and treatment, therefore understanding the QoL-related psychological processes would be important among thyroid patients.

### Social support

Due to a reduced quality of life, patients could need more social support. In general, the most prominent sources of peer support are family, partner or spouse, and friends [53, 54] but a wider range of culture-specific support groups can be available such as church communities, associations, neighbors, or even doctors and health care providers. Studies found that physical and mental health problems are more common in patients who have no family or who live in isolation [53, 55]. Chronic illnesses are a major source of stress, and emotional support from others can facilitate adaptive coping processes, a sense of importance, that they matter to others and that they are worth healing [53, 56]. In addition, social support can also assist patient education: it contributes to the acquisition, processing and understanding of information about the disease and treatment, which in turn can contribute to the maintenance of health-promoting behaviors [53].

### Adherence

Thyroid disorders have many varieties, which require different ways of treatment. The most common type is hypothyroidism, where hormone replacement is needed mostly throughout the life course of the patient [57, 58]. Although synthetic thyroid hormones (a drug commonly used in Hungary is l-thyroxine) are not known to have adverse health effects, a considerable proportion of patients are reluctant to take the prescribed medicines, mostly due to the lack of information [44]. Explaining to them what the medicine they need contains and exactly why they should take it, can usually increase adherence [38, 59]. The complexity of medication can also reduce adherence in hyperthyroidism, as the dosage of the commonly used drugs (methimazole is the most prescribed drug in Hungary) may change during the treatment. Keeping track of all this can be difficult without a proper strategy and help, therefore, it is important to inform patients about their health status to ensure adherence [44, 60, 61]. Indeed, a recent study has shown that non-adherent thyroid patients reported more symptoms of depression, anxiety, and poorer quality of life [61]. Another study on a Belgian sample suggested that hypothyroid patients are not very adherent, but adherence was not correlated with thyroid health [62]. The results are therefore ambiguous in research on thyroid diseases, but results from other chronic diseases suggest that adherence to treatment may play a role in improving quality of life [63–68].

### The aims of the study

The quality of life of patients living with specific chronic diseases is researched in Hungary [4–15]. However, only a few studies have investigated the quality of life of thyroid patients [6, 17], although thyroid disorders affect a considerable proportion of treatment-seeking patients. Exploring the associations between quality of life, adherence, and perceived social support of patients with different thyroid diseases can contribute to a more nuanced understanding of the differences in the psychological characteristics of these patients. In addition to the exploration of group differences (i.e., hypothyroidism [including Hashimoto's thyroiditis] and hyperthyroidism [including Graves' disease]), this study investigates the possible mediating role of social support in the association between adherence and quality of life. Based on the literature [47, 48, 61, 69–73], it is hypothesized that social support will be associated with higher adherence, which in turn is associated with a better quality of life in thyroid patients.

## Methods

### Participants and procedure

The sample consisted adult Hungarian women with specific thyroid disorders. Participants who had not received psychiatric treatment in the last six months or had not received a psychiatric diagnosis in their life were included in the sample. Data collection was conducted using an online questionnaire. Participants were recruited from various social media platforms including thematic Facebook groups dedicated to thyroid patients, Instagram pages, endocrinologists’ webpages, and thyroid disorder clinics' websites. Data collection started on 4th February 2022 and lasted 42 days. Participation in the study was voluntary and anonymous. Respondents were asked to fill out an informed consent form at the beginning of the questionnaire. In total, 1061 respondents completed the online questionnaire; however, two respondents were under the age of 18 years and one respondent provided inconsistent responses (e.g., aged 112 years). The assessment of adherence is relevant only for patients with reported pharmacological treatment; therefore, patients not reporting ongoing pharmacological treatment at the time of the data collection were excluded from further data analysis (*n* = 173). Therefore, the final sample consisted of 885 female patients (M = 35.6 years, SD = 10.7, age range: 18–73 years) with diagnosed thyroid disorders based on self-report (i.e., hypothyroidism [*n* = 824] and hyperthyroidism [*n* = 61]). Demographic characteristics are presented in Table 1.

**Table 1:** Demographic characteristics of the thyroid patients (*N* = 885)

Variables		Hypothyroidism n (%)	Hyperthyroidism n (%)	N (%)
Residence	Capital city	296 (33.5%)	15 (1.6%)	311 (35.1%)
	City	395 (44.6%)	30 (3.4%)	425 (48%)
	Village	133 (15%)	16 (1.8%)	149 (16.8%)
Level of education	No high school diploma	37 (4.1%)	8 (0.9%)	45 (5%)
	High school diploma	271 (30.6%)	32 (3.6%)	303 (34.2%)
	BA, MA degree or higher	516 (58.3%)	21 (2.3%)	537 (60.6%)

**Fig. 1:**
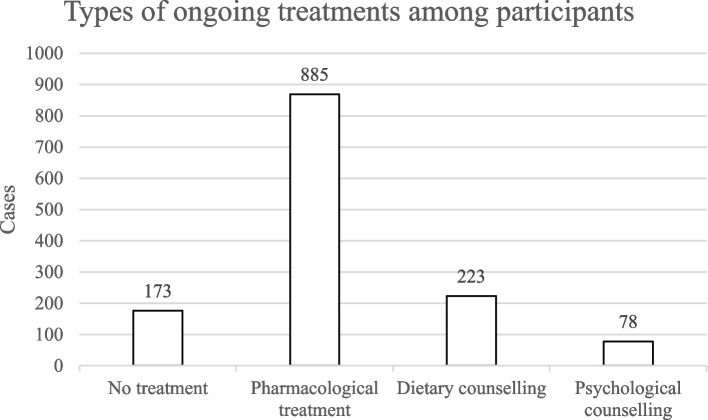
Types of ongoing treatments among participants. No treatment: patients not receiving regular specialist treatment and follow-up from a doctor. Pharmacological treatment: patients receiving medical treatment and follow-up from a doctor. Dietary counseling: patients regularly consulting with a dietician. Psychological counseling: patients regularly consulting with a psychologist

The types of ongoing treatments are presented in Fig. 1. These are overlapping categories; in many cases a patient will receive more than one treatment (13.5% received pharmacological treatment and dietary counselling, 5% received pharmacological treatment and psychological counselling, and 1.3% received all three).

### Measures

First, information was gathered on respondents’ sociodemographic characteristics including their age, residence, education, and the type of thyroid disorders (hypothyroidism, hyperthyroidism, Hashimoto's thyroiditis, Graves’ disease).

The *Thyroid-Related Quality of Life Measure (ThyPRO)* questionnaire measures thyroid-specific quality of life and was validated in Hungarian in 2023 [17]. The original questionnaire contained 85 items and proved to be too long for clinical use, so a shortened version with 39 items was created [74]. An item was included in the ThyPRO assessing an overall quality of life (i.e., “During the past 4 weeks, has your thyroid disease had a negative effect on your quality of life?”), which was used in the present study. This item is rated on a 5-point scale ranging from 0 = ‘Not at all’ to 4 = ‘Very much/Completely’. The item score should be rescaled in the following way: 0 = 0 (no symptoms), 1 = 25, 2 = 50, 3 = 75, 4 = 100 (maximum level of symptoms). For the sake of clarity, scores were reversed in the data analysis; therefore, higher scores reflect higher quality of life.

The *Support Dimension Scale (SDS)* was developed by Caldwell et al. [75]. The Hungarian version was used in this study [76]. The 11-item SDS measures the extent of perceived social support from different persons including parent, child, spouse/partner, relative, schoolmate, helping professional (e.g., doctor), neighbor, church group, co-worker, helping organization, friend. In the present study, a further category “None” was added, which was coded as “0 = “None at all”. Items are rated on a 4-point Likert-scale ranging from 0 = “Not at all” to 3 = “Very much”. Item scores should be summed to get an overall score. Higher scores indicate more peer support.

The *Morisky Medication Adherence Scale* measures patients' cooperation (adherence) in their medication [77]. Higher scores indicate lower adherence. The Hungarian version was used in this study [5, 78]. For the sake of clarity, scores were reversed in the data analysis; therefore, higher scores reflect greater adherence. Cronbach's alpha in the present study: 0.663 which is in line with the literature [79–81].

### Data analysis

SPSS 22 and Mplus 7.4 [82] were used for data analysis. First, based on theoretical considerations [83] two patient groups were created: patients with hypothyroidism (including Hashimoto's thyroiditis, *n* = 824) and patients with hyperthyroidism (including patients with Graves’ disease, *n *= 61). As all study-variables were measured on an ordinal scale, Mann–Whitney U test was conducted for group comparisons. Spearman rank-order correlations were used to explore the associations between study-variables in the two patient groups. Bonferroni correction was applied for multiple testing. Therefore, the *p*-value was set at *p* < 0.01 for group comparisons and correlations. Finally, mediation analysis was performed [84]. As all study-variables was either a single-item variable (i.e., quality of life) or a set of items for which internal consistency is not interpretable (i.e., source of social support), observed variables were included in the mediation model. Therefore, a fully saturated model was estimated with fit indices set to χ2 = 0; *df* = 0, Comparative Fit Index (CFI) = 1.00; Tucker-Lewis Index (TLI) = 1.00; Root-Mean-Square Error of Approximation (RMSEA) = 0.00 by default. A robust weight least squares estimator with mean and variance adjusted statistics (WLSMV) was used, which is appropriate for ordinal variables [85]. Missing data were treated applying listwise deletion, which is the default when using a WLSMV estimator in Mplus. Indirect effects were calculated using 95% bias-corrected bootstrapped confidence intervals based on 10,000 replication samples [86]. Appropriate sample size for the mediation analysis was determined at 377 participants, which allows to detect small-to-medium mediation paths (β = 0.14–0.26) at 0.8 power [87].

## Results

### Descriptive statistics of thyroid patients

The vast majority of the sample consisted of patients with hypothyroidism (*n* = 824; 93.1%, including Hashimoto thyroiditis), while only 6.9% (*n* = 61) of patients reported hyperthyroidism (including Graves’ disease). 31.7% had been living with the disease for 1–4 years, 28.9% for 5–9 years, and 22.1% for 10–19 years. Only a small proportion of patients reported living with the disease for less than 1 year (10%) or for more than 20 years (7.4%). The three most common symptoms patients experienced during their illness were difficulty sleeping (*n* = 702; 79.3%), weakness (*n* = 755; 85.3%) and fatigue (*n* = 861; 97.3%).

### Comparisons between thyroid patient groups

Mann–Whitney U test was conducted to explore possible differences across thyroid patient groups in adherence, quality of life, and social support (see Table 2). No significant difference was found in adherence, quality of life, or social support among the two patient groups.

**Table 2:** Mann–Whitney U tests across thyroid patient groups based on adherence, quality of life, and social support

**Variables**	**Total** (*N* = 885)	**1. Hypothyroidism** (*n* = 824)		**3. Hyperthyroidism** (*n* = 61)		**U**
	*Median*	Mean rank	*Median*	Mean rank	*Median*	
**Adherence**	5.00	435.97	5.00	407.93	5.00	22,993.00
**Quality of life**	50.00	449.09	50.00	360.75	25.00	20,115.00
**Social support**	12.00	442.72	12.00	446.81	12.00	24,899.50

No significant group differences were found at the level of *p* < 0.01 across the comparisons

### Associations between adherence, quality of life, and social support in thyroid patient groups

Spearman rank-order correlations were conducted to explore the associations between adherence, quality of life, and social support in different thyroid patient groups (see Table 3). Higher perceived social support was associated with better life quality in patient groups living with hypothyroidism and hyperthyroidism. These associations were generally weak. Moreover, higher adherence was associated with better life quality in patients with hypothyroidism. However, this association was also weak. Adherence was unrelated to social support in both patient groups. However, the limited sample size could explain the lack of associations with adherence in patients with hyperthyroidism.

**Table 3:** Zero-order correlations between adherence, quality of life, and social support in thyroid patient groups

	1. Adherence	2.Quality of life	3.Social support
Hypothyroidism (*n* = 824)			
1. Adherence	–		
2. Quality of life	**0.16**	–	
3. Social support	0.10	**0.31**	–
Hyperthyroidism (*n* = 61)			
1. Adherence			
2. Quality of life	-0.01	–	
3. Social support	-0.03	**0.37**	–

Boldfaced correlation coefficients are significant at *p* < 0.01. Spearman’s rho correlation coefficients are reported

### Testing the mediator role of social support in the relationship between adherence and quality of life in thyroid patients

In the final step, mediation analysis was performed in which social support was included as a mediator variable between adherence and quality of life. The mediation was performed using the total sample of thyroid patients, irrespective of their disease, as no significant difference was found in key variables between patients in preceding group comparisons. Furthermore, the limited sample size of patients with hyperthyroidism (*n* = 61) did not allow for model construction based on the preceding power calculation (required sample size = 377).

**Fig. 2:**
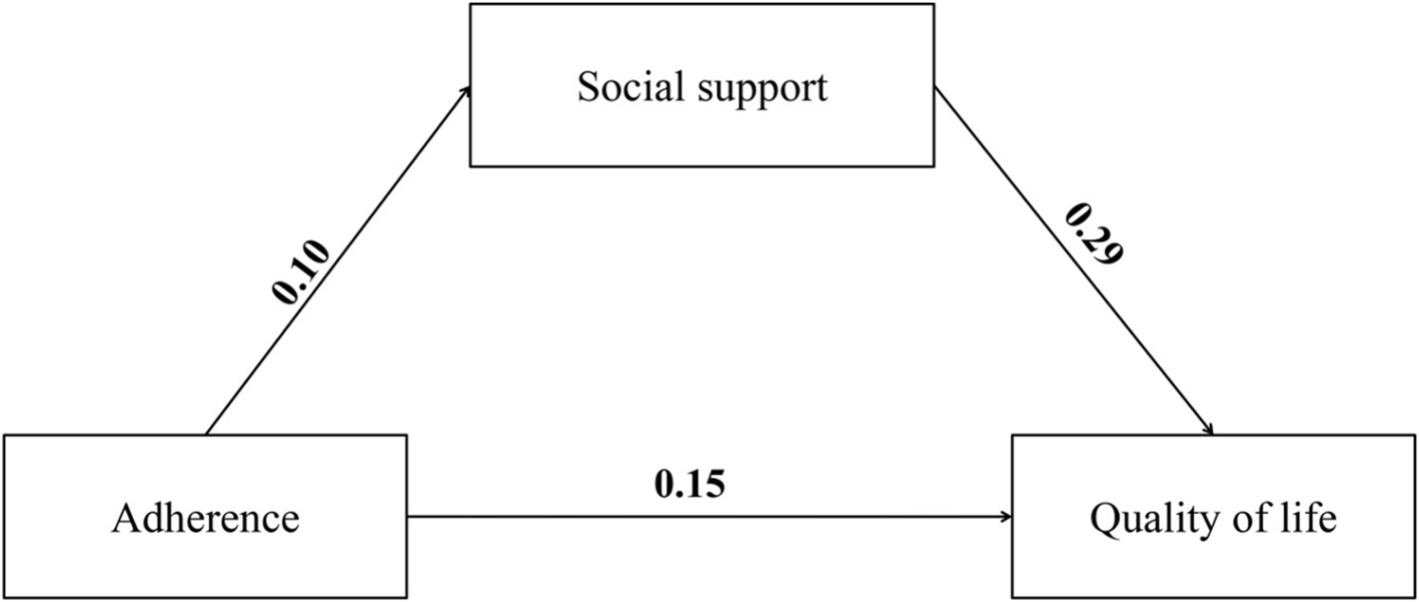
The mediating role of social support in the association between adherence and quality of life among thyroid patients with pharmacological treatment (*n* = 867). Standardized regression coefficients (*β*s) are presented in the figure. Boldfaced paths are significant at *p* < 0.01

Weak, positive associations were found between adherence, social support, and quality of life (see Fig. 2). Greater adherence was associated with higher social support (β = 0.10; 95% CI = 0.03; 0.16]; *p* = 0.004), which in turn was associated with a better quality of life (β = 0.29; 95% CI = 0.23; 0.35]; *p* < 0.001). The direct association between adherence and quality of life was again weak (β = 0.15; 95% CI = 0.08; 0.22]; *p* < 0.001). The total effect was β = 0.18; 95% CI = [0.11; 0.25]; *p* < 0.001, while the total indirect effect was β = 0.03; 95% CI = [0.01; 0.05]; *p* = 0.006. Overall, the model explained only a small proportion of variance of quality of life (11%). Partial mediation was demonstrated.

## Discussion

The present study aimed to explore the quality of life of women with thyroid disorders in Hungary, and to explore possible factors that might have contributed to quality of life in these patients. No substantial differences were found in adherence, social support, and quality of life across patients with different types of thyroid diseases (i.e., hypothyroidism and hyperthyroidism). Weak but consistent associations were found between these factors. Moreover, higher perceived social support partially mediated the relationship between adherence and better quality of life. These findings highlight the importance of supporting mental health care of thyroid patients, as mental and physical health are closely related. Our findings also point out that thyroid patients may not differ considerably in some fundamental psychological resources. The difference in quality of life between the two patient groups was suggested by the literature due to different symptoms of the diseases [57]. However, no substantial difference was found between these patients in terms of adherence, social support, and quality of life. Although the investigated disorders have different symptoms, it seems they have a similar overall negative impact on quality of life [43, 44]. For instance, hypothyroidism is associated with weight gain due to decreased metabolic rate, whereas hyperthyroidism is associated with rapid weight loss. Both processes affect patients' appearance, which can influence psychological well-being and quality of life. Similarly, cold sensitivity in hypothyroidism and increased sweating in hyperthyroidism affect temperature regulation and cause unusual body sensations, can have different effects, but both could reduce quality of life. Moreover, some core symptoms are characteristic to both hyperthyroidism and hypothyroidism such as hair loss, mood instability, anxiety, fatigue, sleep disturbances, or period disorders [24, 57, 58, 88–90]. Therefore, thyroid patients with different types of disorders need increased social support, as symptoms manifest in similar psychological and physical processes (e.g., acceptance of changes in appearance, fertility, and femininity).

Positive association between quality of life and social support was demonstrated, which is consistent with the literature [53, 60, 91]. Higher adherence and better quality of life were also associated, which is also in line with previous findings [92]. However, group differences were found in patients diagnosed with hyperthyroidism: higher levels of adherence and social support were not associated with better quality of life. This contradicts previous findings suggesting that higher social support improves the quality of life. However, it is likely that the small sample size of this patient group does not allow for meaningful comparisons.

Adherence, quality of life, and social support were positively associated in patients with hypothyroidism in this study. Additionally, the relationship between adherence and quality of life was partially mediated by social support. Adherence, amplified by social support, can contribute to the quality of life [53, 93]. The present results suggest that thyroid patients need to mobilize not only their own resources but also their social network to achieve a successful therapy and recover from their illnesses. This systems approach is used in community psychiatric care for people with mental illnesses not only in Hungary [94], but in other countries for the therapy of different chronic illnesses: diabetes [69], polycystic ovary syndrome [70], inflammatory bowel disease [95].

One of the strengths of the present study is that it is among the first studies on the quality of life of thyroid patients in Hungary, using a relatively large sample size of thyroid patients. However, there are some limitations that need to be mentioned. First, the recruitment of respondents was carried out on social media platforms which has been suggested to disproportionately favor more dissatisfied patients, therefore, clinical samples could be a more appropriate population for examining these associations. The present sample is not representative of thyroid patients, and reports of their illnesses were based on self-report which could lead to cross-contamination too between the patient groups. We could not exclude the patients who are receiving special treatments, such as surgery or I131 or who don’t receive treatment at all (e. g. watchful waiting in subclinical cases). Second, the subsample of patients with hyperthyroidism comprised only 61 participants. Therefore, estimations performed on this group is underpowered and clearly explorative. Future research should confirm these results using larger sample sizes. Another important limitation concerns the lack of information on the thyroid function (i.e., TSH levels) of patients, which has been identified as the strongest predictor of life quality among thyroid patients [96]. Due to the self-report nature of the present research, recalling medical information could possibly lead to biased reports, which could raise severe credibility concerns. Nevertheless, the lack of data on TSH levels further undermines the generalizability of the present results. Finally, the study is cross- sectional, which does not allow for clear conclusions regarding causality.

## Conclusion

The quality of life of Hungarian women with thyroid disorders can be affected by the perceived social support, which can strengthen the relationship between adherence and life quality. Patients with thyroid disorders need a socially supportive environment to help them recover, maintain their disorder and their physical and mental health.

### Implication for practice

An important objective of healthcare is to improve the quality of life of chronic patients and contribute to increase life expectancy. It has a special importance not only for patients, their families, and doctors, but has relevance for the broader society [97]. This study found further evidence that adherence is associated with improved quality of life, and social support can contribute to this association. Psychologists can play a key role in increasing adherence by providing patient education and decrease concerns about medication by strengthening the trust in primary healthcare providers. They can also play a role in increasing social support in patients' lives, both in individual sessions and in group therapy for thyroid patients. Finally, mental health professionals can help patients cope with the physical and psychological symptoms of thyroid diseases by applying stress management techniques, improving their confidence and self-esteem, and help them maintain emotional stability. This study underlines the importance of social support in the relationship between adherence and quality of life and contributes to the growing literature of women’s health. The practical relevance of exploring thyroid patients’ mental health is clear: improving patients' quality of life, restoring their sense of control, and developing adaptive coping strategies can take a significant burden off the healthcare system and improve patients' physical and mental health [97].

## Data Availability

The dataset is available from the corresponding author on reasonable request.
